# Minimally Invasive Surgery Versus Conventional Neurosurgical Treatments for Patients with Subcortical Supratentorial Intracerebral Hemorrhage: A Nationwide Study of Real-World Data from 2016 to 2022

**DOI:** 10.3390/diagnostics15111308

**Published:** 2025-05-23

**Authors:** Huanwen Chen, Matthew K. McIntyre, Mihir Khunte, Ajay Malhotra, Mohamed Labib, Marco Colasurdo, Dheeraj Gandhi

**Affiliations:** 1 Department of Neurology, MedStar Georgetown University Hospital, Washington, DC 20007, USA; 2 Neurosurgery, University of Maryland Medical Center, Baltimore, MD 21201, USA; 3 Department of Neurological Surgery, Oregon Health & Science University, Portland, OR 97239, USA; 4 Warren Alpert Medical School, Brown University, Providence, RI 02903, USA; 5 Radiology, Yale New Haven Hospital, New Haven, CT 06510, USA; 6 Interventional Radiology, Oregon Health & Science University, Portland, OR 97239, USA

**Keywords:** intracerebral hemorrhage, intracranial hemorrhage, minimally invasive, neurosurgery, evacuation, real-world, nationwide readmissions database

## Abstract

**Background**: Neurosurgical interventions are often indicated for patients with subcortical, supratentorial intracerebral hemorrhage (ICH); however, the optimal treatment modality is controversial. Whether minimally invasive surgery (MIS) may be superior to conventional craniotomy (CC) or decompressive craniectomy (DC) in real-world clinical practice is unknown. **Methods**: This was a retrospective cohort study of hospitalization data from the 2016–22 Nationwide Readmissions Database. International Classification of Diseases—10th edition (ICD-10) codes were used to identify patients with primary supratentorial subcortical ICH who underwent neurosurgical treatment. Patients with ICH in other brain compartments (other than intraventricular hemorrhage) were excluded. Coprimary outcomes were routine discharge to home without rehabilitation needs (excellent outcome) and in-hospital mortality. Outcomes were compared between MIS versus CC and MIS versus DC, with multivariable adjustments for patient demographics and comorbidities. **Results**: A total of 3829 patients were identified; 418 underwent MIS (10.9%), 2167 (56.6%) underwent CC, and 1244 (32.5%) underwent DC. Compared to CC patients, MIS patients were less likely female (*p* = 0.004) but otherwise had similar patient characteristics; compared to DC patients, MIS patients were older, less likely female, more likely to have mental status abnormalities, more likely to have underlying dementia, less likely to undergo external ventricular drainage, more likely to have vascular risk factors (hypertension, hyperlipidemia, diabetes), and less likely to have underlying coagulopathy (all *p* < 0.05). After multivariable adjustments, MIS patients had higher odds of excellent outcomes compared to CC (OR 1.99 [95%CI 1.06–3.30], *p* = 0.039), and similar odds compared to DC (OR 1.10 [95%CI 0.66–1.86], *p* = 0.73). In terms of in-hospital mortality, MIS had lower odds compared to DC (OR 0.63 [95%CI 0.41–0.96], *p* = 0.032) and similar odds compared to CC (OR 0.81 [95%CI 0.56–1.18], *p* = 0.26). **Conclusions**: For patients with subcortical, supratentorial ICH requiring surgical evacuation, MIS was associated with higherhigher rates of excellent outcomes compared to CC and lower rates of in-hospital mortality compared to DC. However, since key variables such as hematoma size and symptom severity were not available, residual confounding could not be excluded, and results should be interpreted cautiously. Dedicated prospective or randomized studies are needed to confirm these findings.

## 1. Introduction

Spontaneous intracranial hemorrhage (ICH) affects over 3 million patients annually and represents nearly 30% of incident strokes and 50% of stroke-related deaths worldwide [1]. Despite high rates of neurological disability and death, treatment options are limited. More specifically, for patients with supratentorial, subcortical ICH, the role of surgical management is controversial. In 2005, the STICH trial reported negative results for early surgery with conventional craniotomy (CC) versus conservative management for patients with ICH, and subgroup analyses revealed that there may be significant treatment heterogeneity depending on ICH location where surgery may be less effective for subcortical hemorrhages compared to lobar locations [2]. More recently, the SWITCH trial investigated the effectiveness of decompressive hemicraniectomy (DC) in this population [3]; while results were marginally positive, the efficacy of DC was primarily driven by the prevention of extremely poor neurological outcomes (bedridden state or death), and it did not appear to be effective in meaningfully improving the odds of good neurological recovery.

To limit procedural harms associated with surgical ICH treatments, minimally invasive surgery (MIS), which can involve various devices and techniques, has been proposed as a safer alternative to CC or DC [4,5,6]. In 2018, Scaggiante et al. reported in a meta-analysis of randomized trials that MIS techniques may be favorable to conventional treatment including medical management and conventional craniotomy [6]. More recently, in 2024, the randomized controlled MSICH trial results demonstrated that ICH evacuation with MIS techniques (either with endoscopic or stereotactic aspiration) was associated with more favorable clinical outcomes compared with conventional craniotomy, particularly for subcortical hemorrhages [7]. Finally, in 2024, Huan et al. reported in an updated network meta-analysis that MIS, either using endoscopic or minimally invasive puncture approaches, may be superior to conservative management, CC, or DC for patients with ICH [8]. While these data are promising, they largely originate from large academic teaching centers and outcome measurements may be biased from the investigators. Thus, the generalizability of these findings to real-world practice is unknown.

In this retrospective study of nationwide hospitalization records in the United States, we sought to investigate the real-world effectiveness of MIS versus CC and DC for patients with subcortical supratentorial ICH. We hypothesized that MIS would yield superior clinical outcomes compared to CC and DC.

## 2. Methods

### 2.1. Database Characteristics

This was a retrospective analysis of the 2016 to 2022 Nationwide Readmissions Database (NRD), which is part of the Healthcare Cost and Utilization Project (HCUP). The NRD provides information on all hospitalization records of admitted and readmitted patients across 30 geographically dispersed states, representing real-world data across all hospital types and practice settings. In total, NRD captures over 16 million records per year, representing roughly 50% of all hospitalizations in the United States. Patient identifiers are not included in the NRD. As such, this study was exempt from institutional review board approval under the Health Insurance Portability and Accountability Act or informed consent.

### 2.2. Patient Population

All patient diagnoses and procedures were identified using the International Classification of Disease, 10th revision (ICD-10) codes for diagnoses and procedures. Adult patients with a primary diagnosis code for subcortical intracerebral hemorrhage (ICH) who underwent neurosurgical treatment were included; patients with intracranial tumor, subdural hematoma, subarachnoid hemorrhage, ischemic stroke, multi-compartment ICH (except intraventricular hemorrhage) or missing discharge destination data were excluded. Patients were divided into three cohorts: minimally invasive surgery (MIS), conventional craniotomy (CC), and decompressive craniectomy (DC). MIS patients were identified by the presence of ICD-10 codes specifying endoscopic or percutaneous hematoma evacuation, whereas CC was identified by codes specifying open evacuation. DC patients were identified by the presence of codes specifying craniectomy, regardless of the presence of other codes pertaining to hematoma evacuation. Patient demographics (age, sex) were recorded. The presence of intraventricular hemorrhage and the placement of external ventricular drain were identified. Prior anticoagulant use, prior antiplatelet use, and other medical comorbidities (atrial fibrillation, hypertension, hyperlipidemia, diabetes, chronic liver disease, chronic kidney disease, coagulopathy, dementia) were also recorded. Elixhauser comorbidity index was calculated for each patient to estimate the overall medical comorbidity burden [9].

#### 2.2.1. Study Outcomes

The primary outcome is routine discharge to home with self-care, which is a surrogate marker for excellent neurological outcomes [10,11,12]. Other outcomes include discharge to home (with or without in-home care services, a surrogate marker for good outcomes for patients with pre-existing disability [13,14,15]), discharge to a facility (acute rehabilitation, skilled nursing facility, long-term care or any other non-home setting), and in-hospital death (regardless of goals-of-care or hospice services). Other outcomes included hospital length of stay and cost of hospitalization, adjusted for inflation. All ICD-10 codes used for this study were included in Table S1.

#### 2.2.2. Statistical Methods

The number of patients was calculated using discharge-level weights. Patients with missing data were excluded from the analysis. Continuous data were expressed as median and quartiles and compared using Wilcoxon rank-sum tests. Categorical data were represented as percentages and compared using chi-squared tests. Patients treated with MIS were compared to those treated with CC and DC. Multivariable logistic and linear regression models accounting for patient age, sex, intraventricular hemorrhage, external ventricular drain, antithrombotic medication use, captured comorbidities, Elixhauser comorbidity index, and treatment year were used to provide adjusted estimates of differences between MIS and other treatment modalities in terms of discharge outcomes, hospital length of stay, and hospitalization costs. Overall, two-sided *p*-values less than 0.05 were deemed statistically significant. All statistical analyses were performed using R, Version 3.6.2.

## 3. Results

### 3.1. Patient Characteristics

**Figure 1:**
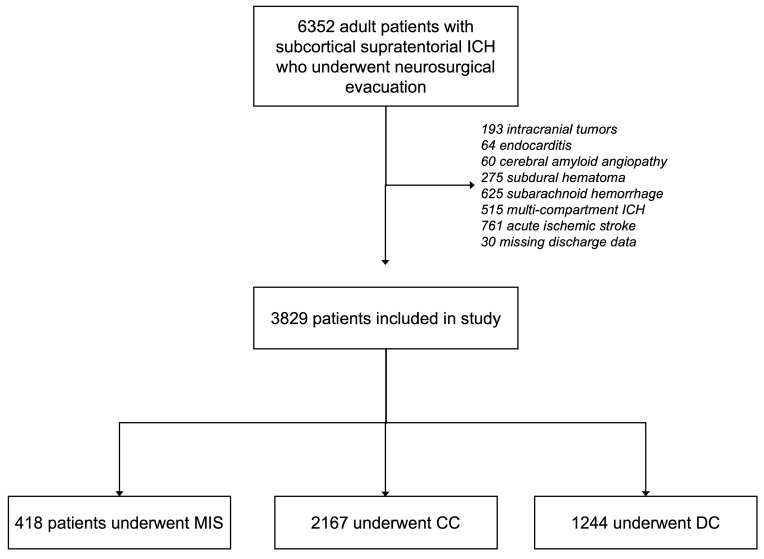
Study flow chart. Abbreviations: ICH—intracerebral hemorrhage; MIS—minimally invasive surgery; CC—conventional craniotomy; DC—decompressive hemicraniectomy.

A total of 6352 patients with subcortical supratentorial ICH who underwent neurosurgical treatment were identified. After excluding 193 patients with intracranial tumors, 64 with endocarditis, 60 with cerebral amyloid angiopathy, 275 with concurrent subdural hematoma, 625 with concurrent subarachnoid hemorrhage, 515 with multi-compartment ICH, 761 with concurrent ischemic stroke, and 30 patients with missing data, 3829 patients were included for analysis. Overall, 418 underwent MIS (10.9%), 2167 (56.6%) underwent CC, and 1244 (32.5%) underwent DC. The study flowchart is presented in Figure 1.

In terms of patient characteristics, MIS patients were less likely female compared to CC patients (27.2% vs. 36.1%, *p* < 0.001) but otherwise had similar patient characteristics (all *p* > 0.05). Compared to DC patients, MIS patients were older (median 58 vs. 52 years, *p* < 0.001), less likely female (27.2% vs. 36.9%, *p* = 0.008), more likely to have underlying dementia (2.9% vs. 0.3%, *p* < 0.001), less likely to undergo external ventricular drainage (25.1% vs. 35.7%, *p* = 0.009), more likely to have vascular risk factors (hypertension, hyperlipidemia, diabetes, all *p* < 0.05), and less likely to have underlying coagulopathy (11.9% vs. 17.1%, *p* = 0.039). All patient characteristics and comparisons are detailed in Table 1.

### 3.2. MIS vs. CC Outcomes

**Figure 2:**
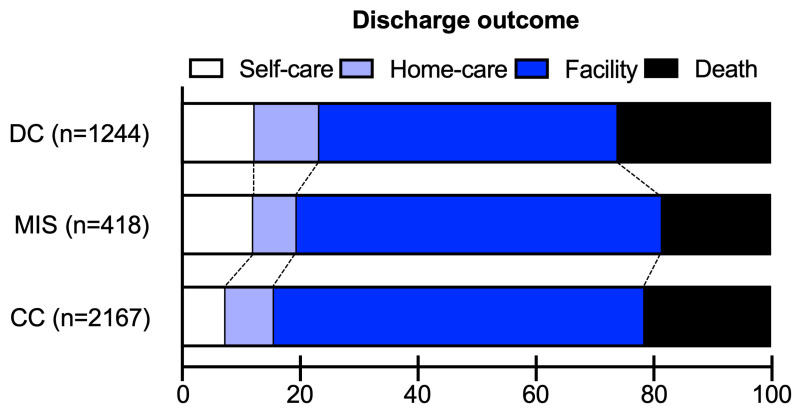
Discharge outcomes of surgical supratentorial subcortical ICHs stratified by treatment modality. Self-care indicates discharge to home with no in-home rehabilitation needs; home-care indicates discharge to home with no in-home rehabilitation services.

In unadjusted analyses, MIS was significantly associated with higher rates of routine discharge compared to CC (12.0% vs. 7.2%, *p* = 0.026; Table 2), and this association remained statistically significant after multivariable adjustments (OR 1.99 [95%CI 1.06–3.30], *p* = 0.039; Table 2). MIS was also associated with higher hospitalization costs than CC (median 89,866 vs. 80,418 USD, *p* = 0.003; Table 2), which also remained statistically significant after multivariable adjustments ( + 10,767 USD [95%CI +702 to +20,831], *p* = 0.03; Table 2). Finally, MIS was associated with longer hospital stays (median 21 vs. 19 days, *p* = 0.042; Table 2); however, this association was no longer statistically significant after multivariable adjustments (*p* = 0.80, Table 2). There were no statistically significant differences between MIS and CC for home discharge or in-hospital death, both before and after multivariable adjustments (all *p* > 0.05, Table 2). Aisual representation of discharge outcomes is presented in Figure 2.

### 3.3. MIS vs. DC Outcomes

In unadjusted analyses, MIS was significantly associated with lower rates of in-hospital death compared to DC (18.5% vs. 26.0%, *p* = 0.026; Table 3), which remained statistically significant after multivariable adjustments (OR 0.63 [95%CI 0.41–0.96], *p* = 0.032; Table 3). There were no statistically significant differences between MIS and DC for routine discharge, home discharge, hospital length of stay, or hospitalization cost, both before and after multivariable adjustments (all *p* > 0.05; Table 3). Visual representation of discharge outcomes are presented in Figure 2.

## 4. Discussion

In this nationwide retrospective study of supratentorial subcortical ICH patients who underwent neurosurgical treatment, we found that MIS evacuation waswas associated with higher rates of favorable neurological outcomes compared to CC, and lower rates of in-hospital death compared to DC. This study provides real-world data suggesting that MIS evacuation may be preferred over conventional neurosurgical treatments for patients undergoing surgery for supratentorial subcortical ICHs.

Our overall finding that MIS was associated with superior outcomes in real-world clinical practice in the United States is consistent with the current literature [6,7,8]. Specifically, when compared to conventional craniotomy, MIS was associated with higher rates of excellent short-term neurological outcomes; this effect may be driven by less iatrogenic injury to healthy brain tissue during surgical exploration. In contrast, MIS was associated with lower rates of in-hospital mortality compared to DC. This may have been driven by an overall less invasive nature of MIS; however, the possible effect of residual confounding where DC patients may have larger and clinically more severe ICHs cannot be excluded.

Of note, while MIS may be preferable to other surgical modalities, whether MIS evacuation is superior to medical management alone is unclear. Due to the lack of information on ICH size and neurological exam within the NRD, it was not feasible to compare medically managed patients to surgical patients as the former would inevitably be associated with milder cases which would confound associations with discharge outcomes. Recently, two trials have explored MIS evacuation of ICH patients compared to conservative management. ENRICH, which compared MIS evacuation (with an endoscopic, trans-sulcal, parafascicular approach) within 24 h to medical management, found a significant treatment benefit associated with MIS evacuation of lobar hemorrhages [16]; however, due to the triggering of a pre-determined adaptation rule, recruitment of anterior basal ganglia hemorrhages was halted early due to lack of observed clinical benefit in this subgroup early in the trial. As such, while the overall results of ENRICH were positive in favor of MIS, the study was underpowered to detect treatment benefits for patients with subcortical ICH. In parallel with ENRICH, the MIND study also sought to investigate the MIS versus medical management for ICH patients, and the study population consisted mostly of subcortical hemorrhages [17]. However, due to the publication of ENRICH, the MIND study was halted early due to concerns regarding equipoise, which also compromised its statistical power. Thus, overall, there is currently a lack of high-level clinical trial data on MIS for the treatment of subcortical supra-tentorial ICHs. Future dedicated trials of MIS for subcortical ICHs compared to medical management are needed to further demonstrate its effectiveness.

Another interesting finding in our study was that MIS was associated with significantly increased cost of hospitalization compared to conventional craniotomy. The more specialized procedure and associated equipment may likely be driving this difference between MIS and CC. Future studies are needed to investigate the cost-effectiveness of MIS for ICH. More importantly, the higher costs of MIS may limit the accessibility of this treatment in rural or socio-economically challenged locales. Future studies are also needed to identify potential discrepancies in access to MIS treatment across the United States and worldwide, especially considering recent positive results from ENRICH and MIND trial results for lobar hemorrhages [16,17]. One possible bottleneck for access to MIS treatments may be the limited neurosurgery workforce in the United States [18]. Given recent increases in the neuro-interventional workforce as a result of the advent of stroke thrombectomy and chronic subdural hematoma treatments [19,20,21,22,23], it has been suggested that, with appropriate procedural training, MIS evacuation could be performed by neurointerventionalists [24]. Future efforts are needed to assess whether MIS evacuation of ICHs can be safely performed by neurointerventionalists.

### Limitations

Our study has several limitations. First, as a retrospective analysis of a large administrative database, we were unable to obtain disease-specific measures such as hemorrhage volume, radiographic features, ICH score, clinical exam, and other hidden/unmeasured confounders [25,26,27]. As such, our analysis was limited to only surgical patients, and comparison with medically managed patients was not feasible. Importantly, lack of information on hematoma size is a major limitation, as this factor may have introduced significant confounding by indication (larger hematomas may be associated with CC/DC and therefore worse outcomes). Future prospective or randomized studies are needed to confirm our study findings. Second, while discharge destinations can be used as a surrogate measure of neurological outcomes following cerebrovascular events, more dedicated long-term outcomes such as modified Rankin scale [28] and patient quality of life [29,30] are not available. Furthermore, a majority of patients were discharged to a facility; however, information on the type of facility (e.g., acute rehabilitation, nursing home, hospice care, etc.) is not reported in the NRD. Third, the study period is limited to 2016 to 2022 due to data availability, which predates the publication of ENRICH and the presentation of MIND, which may have impacted current clinical practice. Future studies are needed to further confirm our study findings in more contemporary settings.

## 5. Conclusions

In this retrospective study of nationwide real-world hospitalization data in the United States, MIS was associated with higher rates of excellent outcomes compared to CC and lower rates of in-hospital mortality compared to DC for patients with subcortical, supratentorial ICH requiring surgical evacuation. However, since key variables such as hematoma size and symptom severity were not available, residual confounding could not be excluded, and results should be interpreted cautiously, Dedicated prospective or randomized studies are needed to confirm these findings.

## Figures and Tables

**Table 1:** Patient characteristics.

	Total	MIS	CC	DC	*p*-Values	
Characteristic—Median (Q1–Q3) or % (n)	N = 3829	N = 418	N = 2167	N = 1244	MIS vs. CC	MIS vs. DC
Age (years)	55 (45–64)	58 (49–65)	56 (47–65)	52 (42–62)	0.34	<0.001 *
Female sex	35.4% (1356)	27.2% (114)	36.1% (783)	36.9% (459)	0.004 *	0.008 *
Intraventricular extension	31.8% (1217)	32.2% (135)	31.2% (676)	32.6% (406)	0.79	0.92
External ventricular drain	28.5% (1093)	25.1% (105)	25.1% (543)	35.7% (445)	1.00	0.009 *
Antithrombotic medications						
Anticoagulant use	6.1% (233)	4.0% (17)	6.1% (133)	6.7% (83)	0.20	0.15
Antiplatelet use	2.0% (78)	0.7% (3)	2.4% (51)	1.9% (24)	0.087	0.15
Comorbidities						
Atrial fibrillation	8.6% (328)	7.1% (30)	9.8% (211)	7.0% (87)	0.18	0.93
Hypertension	80.1% (3068)	83.8% (350)	83.8% (1817)	72.4% (901)	0.99	<0.001 *
Hyperlipidemia	26.4% (1010)	29.7% (124)	28.6% (620)	21.4% (266)	0.74	0.015 *
Diabetes	23.2% (890)	24.9% (104)	25.8% (559)	18.2% (227)	0.78	0.013 *
Chronic liver disease	5.3% (203)	5.6% (23)	4.8% (105)	6.1% (75)	0.61	0.75
Chronic kidney disease	17.0% (653)	16.3% (68)	17.7% (384)	16.1% (200)	0.60	0.94
Coagulopathy	15.0% (574)	11.9% (50)	14.4% (312)	17.1% (212)	0.31	0.039 *
Dementia	1.7% (63)	2.9% (12)	2.2% (47)	0.3% (4)	0.57	<0.001 *
Elixhauser comorbidity index	16 (11–21)	15 (11–21)	16 (10–21)	17 (10–23)	0.71	0.41
Treatment year						
2016	9.1% (349)	11.3% (47)	11.3% (245)	4.6% (57)	0.40	0.004 *
2017	11.5% (440)	13.5% (56)	11.7% (254)	10.5% (130)		
2018	13.1% (501)	9.2% (38)	14.7% (319)	11.5% (143)		
2019	16.6% (636)	17.4% (73)	16.6% (360)	16.3% (203)		
2020	17.1% (653)	19.1% (80)	14.7% (318)	20.5% (255)		
2021	17.4% (667)	12.4% (52)	16.8% (364)	20.2% (252)		
2022	15.2% (583)	17.2% (72)	14.1% (306)	16.4% (205)		

Bold and * denotes statistical significance (*p* < 0.05) for emphasis.

**Table 2:** MIS vs. CC outcomes.

	Unadjusted Comparisons			With Multivariable Adjustments	
Outcome	MIS (n = 418)	CC (n = 2167)	*p*-value	OR or B [95%CI]	*p*-Value
Routine discharge	12.0% (50)	7.2% (157)	0.026 *	1.99 [1.06 to 3.30]	0.039 *
Home discharge	19.3% (81)	15.5% (336)	0.16	1.35 [0.94 to 2.00]	0.097
In-hospital mortality	18.5% (77)	21.4% (464)	0.30	0.81 [0.56 to 1.18]	0.26
Length of hospital stay (days)	21 (13–37)	19 (11–34)	0.042 *	0.43 [−2.91 to 3.77]	0.80
Cost of hospitalization (USD)	89,866 (60,656–139,896)	80,418 (52,437–123,765)	0.003 *	10,767 [702 to 20,831]	0.036 *

Bold and * denotes statistical significance (*p* < 0.05) for emphasis.

**Table 3:** MIS vs. DC outcomes.

	Unadjusted Comparisons			With Multivariable Adjustments	
Outcome	MIS (n = 418)	DC (n = 1244)	*p*-Value	OR or B [95%CI]	*p*-Value
Routine discharge	12.0% (50)	12.2% (152)	0.94	1.10 [0.66 to 1.86]	0.73
Home discharge	19.3% (81)	23.2% (289)	0.23	0.82 [0.54 to 1.25]	0.35
In-hospital mortality	18.5% (77)	26.0% (323)	0.026 *	0.63 [0.41 to 0.96]	0.032 *
Length of hospital stay (days)	21 (13–37)	19 (9–36)	0.077	2.10 [−1.59 to 5.80]	0.26
Cost of hospitalization (USD)	89,866 (60,656–139,896)	88,000 (56,766–144,319)	0.9	2984 [−10,045 to 16,014]	0.65

Bold and * denotes statistical significance (*p* < 0.05) for emphasis.

## Data Availability

Data used in this study are publicly availbe for purchase at https://hcup-us.ahrq.gov/.

## Supplementary Materials

The following supporting information can be downloaded at https://www.mdpi.com/article/10.3390/diagnostics15111308/s1, Table S1: ICD-10 codes.
