# Supplementary material for: Minimally Invasive Surgery Versus Conventional Neurosurgical Treatments for Patients with Subcortical Supratentorial Intracerebral Hemorrhage: A Nationwide Study of Real-World Data from 2016 to 2022
Source: Diagnostics (Basel). 2025 May 23;15(11):1308. doi: 10.3390/diagnostics15111308 (PMC12154345; doi:10.3390/diagnostics15111308)
Supplement: Supplementary file 1 [file diagnostics-15-01308-s001.zip › BG ICH Table S1.pdf]

Supplementary Table S1: ICD-10 codes used for study

	**ICD-10 codes**
**Inclusion**	
Supratentorial subcortical ICH (top diagnosis code)	I61.0
**Exclusion**	
Multicompartment ICH	Any I61.x other than I61.0 and I61.5
Intracranial tumor	C70, C71, D33, C72, R90.0
Subdural hemorrhage	I62
Subarachnoid hemorrhage	I60
Ischemic stroke	I63
Endocarditis	*I33*
Cerebral amyloid angiopathy	*I68.0*
**Treatments**	
Minimally invasive surgery	*009040Z, 00904ZZ, 00C040Z, 00C04ZZ, 009030Z, 00903ZZ, 00C030Z, 00C03ZZ, 009740Z, 00974ZZ, 00C740Z, 00C74ZZ, 009730Z, 00973ZZ, 00C730Z, 00C73ZZ, 009840Z, 00984ZZ, 009940Z, 00994ZZ, 00C840Z, 00C84ZZ, 00C940Z, 00C94ZZ, 009830Z, 00983ZZ, 009930Z, 00993ZZ, 00C830Z, 00C83ZZ, 00C930Z, 00C93ZZ*
Conventional craniotomy	*009000Z, 00900ZZ, 00C000Z, 00C00ZZ, 009700Z, 00970ZZ, 00C700Z, 00C70ZZ, 009800Z, 00980ZZ, 009900Z, 00990ZZ, 00C800Z, 00C80ZZ, 00C900Z, 00C90ZZ*
Decompressive hemicraniectomy	*00N00, 00N70, 00N10, 00N20, 00B70, 00B00, 00B10, 00B20, 0N50, 0N51, 0N53, 0N54, 0N55, 0N56*

**Covariables**	**ICD-10 codes**
EVD	0096
IVH	I61.5
AC use	Z79.01
AP use	Z79.02, Z79.82
Atrial Fibrillation or Flutter	I48.0, I481.1, I48.19, I48.20, I48.21, I48.3, I48.4, I48.91, I48.92

All other ICD-10 codes were derived from Quan et al. (reference #9).
